# Survey and Molecular Characterization of Sarcocystidae protozoa in Wild Cricetid Rodents from Central and Southern Chile

**DOI:** 10.3390/ani13132100

**Published:** 2023-06-24

**Authors:** Pablo Oyarzún-Ruiz, Richard S. Thomas, Adriana M. Santodomingo, Juan E. Uribe, Marlon M. Ardila, Diana M. Echeverry, Sebastián Muñoz-Leal, María C. Silva-de la Fuente, Marco Loyola, Cristina J. Palma, Carlos Landaeta-Aqueveque, AnaLía Henríquez

**Affiliations:** 1 Facultad de Ciencias Veterinarias, Universidad de Concepción, Chillán 3812120, Chile; pablooyarzunruiz@gmail.com (P.O.-R.); richardthomasshz@gmail.com (R.S.T.); adrianasantodomingo@gmail.com (A.M.S.); marlonardila@udec.cl (M.M.A.); sebamunoz@udec.cl (S.M.-L.); crispalma@udec.cl (C.J.P.); 2 Departamento de Biodiversidad y Biología Evolutiva, Museo Nacional de Ciencias Naturales (MNCN-CSIC), 28006 Madrid, Spain; juanuribearboleda@gmail.com; 3 Facultad de Ciencias Básicas, Universidad del Atlántico, Puerto Colombia 81007, Colombia; 4 Facultad de Ciencias de la Naturaleza, Universidad San Sebastián, Concepción 4080871, Chile; diana.echeverry@uss.cl (D.M.E.); marco.loyola@uss.cl (M.L.); alhenriquezh@gmail.com (A.H.); 5 Facultad de Ciencias Agrarias y Forestales, Universidad Católica del Maule, Curicó 3340000, Chile; msilva@ucm.cl

**Keywords:** molecular characterization, *Abrothrix*, Coccidia, Cricetidae, *Besnoitia*, phylogeny, Sarcocystinae, *Sarcocystis*, Toxoplasmatinae

## Abstract

**Simple Summary:**

Sarcocystidae is a family of protozoa whose life cycles usually include two hosts: an intermediate or paratenic host, where protozoa form tissular cysts, and a definitive host, whose intestines usually become infected after consuming tissular cysts. In Chile, studies of sarcocystids have been performed mostly on domestic animals, and scarce data are available from wildlife. A total of 207 rodents, encompassing six native species, from 13 localities in Central and Southern Chile, were studied to assess the presence of Sarcocystidae. DNA was isolated from several tissues and phylogenetic analyses were performed. Blood smear examinations and histological studies of several organs were performed. Overall, three individuals of rodents were PCR-positive and three different genotypes were retrieved belonging to *Sarcocystis*, *Besnoitia*, and Toxoplasmatinae protozoa. No protozoa were found during microscopic examinations. Although it was not possible to confirm whether the findings corresponded to parasitism or accidental encounter, the phylogenetic positions of the genotypes support the hypothesis that they are parasites of rodents. All three genotypes are suggested as potential new taxa in the Sarcocystidae family.

**Abstract:**

In Chile, studies of parasites from the family Sarcocystidae (Apicomplexa) have mostly been related to domestic animals. We aimed to assess the presence of Sarcocystidae taxa in cricetid rodents from Central and Southern Chile. We studied 207 rodents, encompassing six species, from 13 localities. We isolated DNA from tissue samples, amplified the Sarcocystidae 18S rRNA gene with polymerase chain reaction, and performed phylogenetic analyses using maximum likelihood and Bayesian inferences. In addition, we examined blood smears and performed histological studies in organs from Sarcocystidae DNA-positive animals. Three specimens were DNA-positive and three genotypes were retrieved and named: *Sarcocystis* sp. P61, related to *Sarcocystis strixi*, was detected in two *Abrothrix olivacea*. Toxoplasmatinae gen. sp. P99 was retrieved from those same two specimens, and was related to *Toxoplasma* and other genera, although it branched independently. *Besnoitia* sp. R34 was detected in one *Abrothrix hirta*, and was clustered with congeneric species associated with rodents. No protozoa were found during microscopic studies; thus, it was not possible to confirm parasitic interactions rather than accidental encounters. However, the close relatedness of the retrieved genotypes to parasites of rodents supports the hypothesis of host–parasite associations. All three genotypes are suggested as potential new taxa, including a putative new genus.

## 1. Introduction

Coccidia are a group of parasites that belong to the *phylum* Apicomplexa and are commonly found in a variety of hosts, including wild rodents [1]. These parasites can be transmitted to other animals, including humans, through the consumption of oocysts shed through contaminated feces or parasitic cysts present in the host’s body [1,2].

The Sarcocystidae family includes more than 350 nominal species, with the genus *Sarcocystis* considered the most specious. Some species of the family, such as *Toxoplasma gondii*, *Cystoisospora belli*, and *Neospora caninum*, are of medical and veterinary importance [1]. The life cycle is heteroxenous in most species, with predators acting as definitive hosts and prey species acting as intermediate or paratenic hosts [1,2].

Phylogenetic analyses split the family Sarcocystidae into four main clades. The subfamily Toxoplasmatinae corresponds to clade A, encompassing the genera *Hyaloklossia*, *Cystoisospora*, *Besnoitia*, *Hammondia*, *Toxoplasma*, and *Neospora*. The subfamily Sarcocystinae, represented by genus *Sarcocystis*, encompasses three clades: B, which mostly infects birds; C, which infects humans, felids, and canids; and D, which infects snakes as definitive hosts [1,3]. In South America, parasites of the Sarcocystidae family have been detected in rodents and marsupials from Argentina, Brazil, and Peru [4,5,6,7,8,9].

In Chile, there are 73 species of rodents, including native and exotic species that inhabit different ecosystems, and most belong to the Cricetidae family [10]. However, despite being considered the richest order of mammals in Chile [10], studies on their parasites have mostly focused on helminth and arthropod fauna [11,12], with few studies on their protozoa [8,13,14], including *Trypanosoma cruzi* [15]. Thus, to the best of our knowledge, *Sarcocystis muris* is the only report of a sarcocystid species parasitizing introduced rodents, *Rattus* sp. (Muridae), in the country [16], and there are no reports of sarcocystids in cricetid rodents. The purpose of the present study was to assess the presence of Sarcocystidae species in cricetid rodents of Central and Southern Chile and their phylogenetic position in the family.

## 2. Materials and Methods

**Figure 1:**
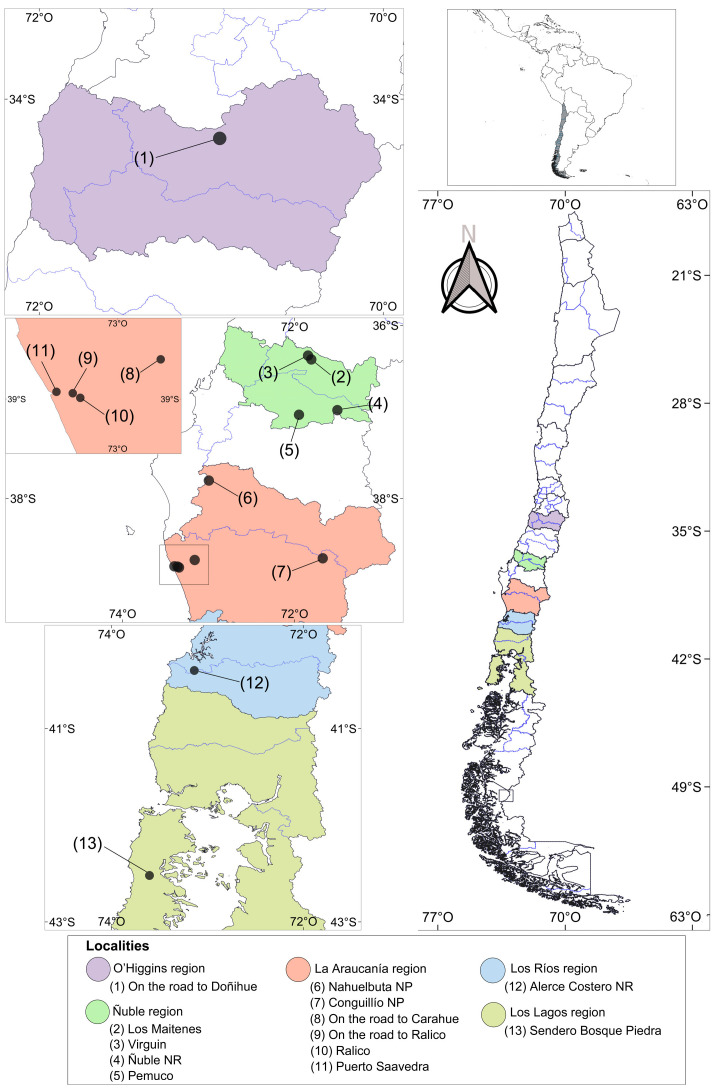
Map of the locations and their relationship with Chile and South America.

Wild cricetid rodents were collected from 13 localities in Central and Southern Chile between 2017 and 2019 (Figure 1; Table S1). Trapping and euthanasia were performed as previously described [17]. In short, live traps (Sherman and Rodentraps) were used to catch rodents in five-night campaigns at each location; rodents were killed with isoflurane overdose according to the AVMA Guidelines for the Euthanasia of Animals 2020. Samples of most organs were kept in ethanol 95%, with the exception of a part of the diaphragm, the quadriceps femoris, and half of the brain, which were kept in formalin 3.6% (p/v). The captured number of specimens per species was determined in accordance with the capture permits given by Chilean agencies; this number was also limited by trapping success and captures were performed in accordance with Chilean laws [18]. None of the rodent species analyzed in this study were of conservation concern. Taxonomic identification of the rodents was performed following the literature [19,20,21]. Genomic DNA was extracted from liver and spleen samples using the DNeasy Blood & Tissue Kit (QIAGEN^®^, Hilden, Germany). A conventional polymerase chain reaction (PCR) was then implemented to amplify a fragment of the mammal glyceraldehyde-3-phosphate-dehydrogenase (*GAPDH*) gene as internal control [22]. The *GAPDH*-positive samples were screened for the Apicomplexa 18S rRNA gene using the protocols from Oosthuizen et al. [23] and Greay et al. [24]. PCRs were carried out using a thermal cycler (Applied Biosystems, Thermo Fisher Scientific, Waltham, MA, USA) with a reaction mixture consisting of 12.5 μL of PCR Master Mix, 1 μL of each primer, and 8.5 μL of DNase-free water. DNA of *Babesia canis* and DNase-free water were used as a positive control and negative control, respectively. Amplicons of the expected size were purified and sequenced in both directions at Macrogen (Seoul, Republic of Korea). The obtained sequences were quality controlled and assembled with Geneious Prime version 2021.2.2 (Biomatters Ltd., Boston, MA, USA, https://www.geneious.com) and subsequently compared to previous sequences using the BLASTn tool. The newly determined sequences plus the homologs from GenBank were aligned with MAFFT using the FFT-NS-Ix1000 algorithm [25]. Informative regions were extracted from the alignments using BMGE [26], using default parameters. The phylogenetic relationships were assessed using the maximum likelihood (ML) [27] and Bayesian inference (BI) [28,29] with IQ-TREE-v-1.6.1 (http://www.iqtree.org/) [30] and MrBayes-v-3.2.6 (https://nbisweden.github.io/MrBayes/index.html) [31], respectively. The best-fit evolutionary models were calculated with ModelFinder [32] for ML inference using the “-m MFP-mrate G” command. For the BI, the “lset nst = mixed rates = gamma” option was incorporated in the MrBayes command line [33]. The Bayesian Information Criterion was used to select the best-fit models. A sub-dataset, including *Goussia pannonica* as an outgroup, was used to assess the corrected pairwise genetic distance between the species representing each genus of clade A of the family Sarcocystidae. The corrected pairwise genetic distance was calculated using raxmlGUI [34,35] for RAxML [36] under the GTR + GAMMA + I substitution model.

Tissue samples of muscle and brain of Sarcocystidae DNA-positive rodents, i.e., those that yielded amplicons of the Apicomplexa 18S rRNA gene, were histopathologically examined to assess the presence of cysts with an optical microscope (Leica DM 1000, Wetzlar, Germany). This examination was performed using tissue cuts processed in an automatic processor. Then, 4 µm paraffin sections were stained with hematoxylin and eosin stain. Overall, 10 cuts per sample were examined under an optical microscope to enhance the likelihood of finding cysts if they existed. Samples of muscle and brain were used because the initial extraction and fixation of the tissues were carried out for other studies searching for other pathogens [17], and the same tissues submitted to PCR were not preserved for histopathological studies. In addition, blood smear samples of the same rodents were stained with Giemsa and examined with a microscope (stated above) for the presence of blood forms such as tachyzoites.

## 3. Results

Overall, 207 rodents belonging to six species and four genera were captured (Table S1). All PCRs targeting the *GAPDH* gene yielded expected-size amplicons, confirming successful DNA extraction. Sarcocystidae DNA belonging to three different genotypes were found in three samples (1.44%).

Two genotypes were found in two specimens of *Abrothrix olivacea* (Cricetidae) from Chiloé and were named *Sarcocystis* sp. P61 and *Toxoplasmatinae gen.* sp. P99. The *Sarcocystis* sp. P61 genotype shared 99.93% identity (1484/1485, 100% query cover, 0 gaps, 0 E-value) with *Sarcocystis strixi* (MF162315), which was detected in *Strix varia* (Strigidae) in the USA [37], while the Toxoplasmatinae gen. sp. P99 genotype showed variable similarities (98.48–98.75%) to different species within the family Sarcocystidae, such as *Hammondia heydorni* (JX220987), *Hammondia triffittae* (GQ984222), *N*. *caninum* (GQ899206), *T gondii* (EF472967), and *Besnoitia besnoiti* (AY833646). Finally, the third genotype, herein named *Besnoitia* sp. R34, was found in an *Abrothrix hirta* (Cricetidae) from Alerce Costero National Park, and it shared 99.93% of its identity (1494/1495, 100% query cover, 0 gaps, 0 E-value) with *Besnoitia jellisoni* (AF291426) culture-derived zoite sequences in the USA [38].

**Figure 2:**
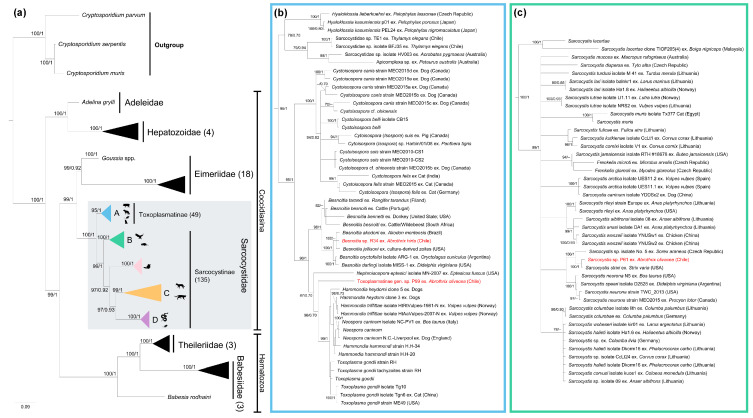
Consensus tree inferred from a subset of Apicomplexan species using maximum likelihood (ML) and Bayesian inference (BI) methods. The tree is based on 216 sequences of the 18S rRNA gene and an alignment of 1742 base pairs. The best-fit evolutionary models for ML and BI were TPM3 + F + G4 and *M*_85_, *M*_177_, *M*_147_, *M*_15_, *M*_179_, respectively. Ultrafast-bootstrap values and Bayesian posterior probabilities are indicated above or below each branch, following Minh et al. [39] and Huelsenbeck and Rannala [33]. (**a**) Overall phylogeny encompassing several families within Apicomplexa. Numbers in parentheses after the family and subfamily names refer to number sequences in each clade. Clades A, B, C, and D represent the different final host groups for the Sarcocystidae family using Doležel et al. [3] classification. Domestic and synanthropic animals (clade A), small mammals and birds (clade B), canids and felids (clade C), and snake–rodent species (clade D). The sequences characterized in the present study are highlighted in red font. (**b**) Subfamily Toxoplasmatinae. (**c**) Subfamily Sarcocystinae. Silhouettes of animals were downloaded from the PhyloPic portal (https://www.phylopic.org/ (accessed on 22 May 2023)).

The best-fit evolutionary models for ML and BI were TPM3 + F + G4 and M85, M177, M147, M15, M179, respectively. The tree and the ultrafast-bootstrap values and Bayesian posterior probabilities are indicated in Figure 2, following the criteria of Minh et al. [39] and Huelsenbeck and Rannala [33]. The phylogenies placed our genotypes into two clades of the family Sarcocystidae, with *Besnoitia* sp. R34 and *Toxoplasmatinae gen.* sp. P99 in clade A, and *Sarcocystis* sp. P61 in clade B (Figure 2). *Besnoitia* sp. R34 formed a monophyletic clade close to *Besnoitia* sequences from American small mammals (Figure 2b), while *Sarcocystis* sp. P61 clustered with *Sarcocystis* sequences isolated from rodents and birds (Figure 2c). On the other hand, Toxoplasmatinae gen. sp. P99 branched independently within the subfamily Toxoplasmatinae (Figure 2b), and the corrected pairwise genetic distances were always above 1% (see Table S2). The new GenBank accession numbers and those used for phylogenetic analyses are listed in Supplementary Table S3. No protozoa were found in the histological and blood smear samples.

## 4. Discussion

To the best of our knowledge, this study represents the first molecular characterization of sarcocystid protozoa DNA obtained from Chilean native rodents, and the first record of these taxa in the genus *Abrothrix*, which is endemic to South America [21]. Previously, the only available sequences of sarcocystid protozoa in Chilean micromammals were those provided by Merino et al. [6] and Santodomingo et al. [8]. However, the lack of histological and blood observations of these protozoa renders it difficult to confirm that the findings corresponded to parasitism rather than accidental encounters. Despite this fact, the molecular evidence supports the idea that these parasites are present in the studied area, either in the examined rodents or in other host species.

According to the phylogenies presented herein, *Sarcocystis* sp. P61 was closely related to *S. strixi*, a species originally described in *S. varia* as its definitive host, and experimentally infected KO mice were identified as laboratory intermediate hosts [37] (Figure 2b). This fact suggests that *Sarcosystis* sp. P61 could cycle among owls and rodents, supporting the hypothesis that the findings of the present study could correspond to a host–parasite interaction rather than an accidental encounter. Further investigations should test the host–parasite interaction. For instance, an examination of *Strix rufipes*—the only *Strix* species reported in Chile [40], and which predates *A. olivacea* [41]—for the presence of *Sarcocystis* sp. P61 could unveil a definitive host for this putative new taxon of the Sarcocystidae family.

The genus *Besnoitia* has 10 nominal species, although it is mostly known due to *B. besnoiti*, which parasitizes cattle and results in important economic losses [42]. Parasites of this genus have been recorded in different species of large and small mammals, which act as intermediate hosts, although some records have also been found in reptiles [42,43]. Our isolate (*Besnoitia* sp. R34) was positioned into a clade of *Besnoitia* species associated with rodents, marsupials, and lagomorphs [44]. This genotype formed a well-supported clade with *B. jellisoni*, a species that uses North American rodents as intermediate hosts [43,45], and *Besnoitia akodoni*, which was isolated from a South American rodent [5]. This evidence suggests a specific clade associated with rodents, supporting the hypothesis of a host–parasite interaction rather than an accidental finding. Histopathological studies have found *Besnoitia* sp. and *B. jellisoni*-like cysts in the reproductive tissue of adult rodents, and in the fetal tissues of *Lagostomus maximus* (Chinchillidae) and *Dipodomys* sp. (Heteromyidae), suggesting vertical transmission [4,44], as also occurs in other Toxoplasmatinae, e.g., *T*. *gondii* and *N*. *caninum* [46,47,48]. Future studies should consider sampling additional tissues, such as those mentioned above, and should not only target *Besnoitia*, but also identify other sarcocystids that may invade these tissues.

An undetermined taxon, Toxoplasmatinae gen. sp. P99, was isolated in one specimen of *A*. *olivacea*. With high statistical support, this genotype branched separately from the genus *Nephroisospora* and a clade encompassing the genera *Neospora*, *Hammondia*, and *Toxoplasma*. *Nephroisospora* is known to parasitize bat kidneys [49]. On the other hand, *Toxoplasma*, *Neospora*, *Besnoitia*, and *Hammondia* parasitize several vertebrates, including rodents [50,51,52], which serve as intermediate or paratenic hosts [1]. Despite this fact, Toxoplasmatinae gen. sp. P99 is phylogenetically separated from all known genera within the Sarcocystidae family. Therefore, it is likely that this genotype could represent a novel species and even a new genus within this family (see Figure 1 and Table S2). While the ecology and evolutionary relationships of this genotype are still unclear, additional surveys of rodents and potential carnivorous hosts would help to validate its identity.

## 5. Conclusions

The presence of sarcocystid DNA in the Chilean wild rodents *A*. *olivacea* and *A*. *hirta*, as well as its phylogenetic closeness to taxa of the Sarcocystidae family that parasitize rodents, suggests that the sarcocystid genotypes reported herein could parasitize *A. olivacea* and *A. hirta*. In addition, the sarcocystid genotypes reported in this work could represent potential new taxa within this family, including a putative new genus.

## Data Availability

The data presented in this study are available in Supplementary Tables S1–S3.

## Supplementary Materials

The following supporting information can be downloaded at: https://www.mdpi.com/article/10.3390/ani13132100/s1, Table S1: Collection localities with the geographical coordinates and rodent species caught; Table S2: Overview of the corrected pairwise genetic distance between species representing each genus of the Toxoplasmatinae subfamily (clade A; see Figure 1), as based on the alignment of 718 base pairs (bp) of 22 sequences of the 18S rRNA gene; Table S3: GenBank accession numbers for the sequences used for Sarcocystidae phylogeny reconstruction based on the 18S rRNA gene.
