# Supplementary material for: Survey and Molecular Characterization of Sarcocystidae protozoa in Wild Cricetid Rodents from Central and Southern Chile
Source: Animals (Basel). 2023 Jun 24;13(13):2100. doi: 10.3390/ani13132100 (PMC10340073; doi:10.3390/ani13132100)
Supplement: Supplementary file 1 [file animals-13-02100-s001.zip › Table S2. Overview table of corrected pairwise genetic distance from Toxoplasmatinae subfamily.pdf]

Table S2. Overview table of corrected pairwise genetic distance between species representing each genus of the Toxoplasmatinae subfamily (clade A, see Figure 1A), based on the alignment of 718 base pairs (bp) of 22 sequences of the 18S rRNA gene.

	Hyaloklossia sp.	Sarcocystidae sp.[a]	Sarcocystidae sp.[b]	Apicomplexa sp.[b]	*Cystoisospora* sp.	*Besnoitia* sp.	*Nephroisospora eptesici*	Toxoplasmatinae gen. sp. P99	*Neospora caninum*	*Hammondia* sp.	*Hammondia hammondi*	*Toxoplasma gondii*
Hyaloklossia sp.	0.0038											
Sarcocystidae sp.[a]	0.0537	N/A										
Sarcocystidae sp.[b]	0.0371	0.0479	N/A									
Apicomplexa sp.[b]	0.0866	0.1588	0.0609	N/A								
Cystoisospora sp.	0.0354	0.0522	0.0473	0.1360	0.0112							
Besnoitia sp.	0.0366	0.0641	0.0497	0.1330	0.0306	0.0126						
Nephroisospora eptesici	0.0488	0.0699	0.0582	0.1451	0.0359	0.0349	N/A					
Toxoplasmatinae gen. sp. P99	0.0456	0.0604	0.0359	0.1389	0.0320	0.0294	0.0371	N/A				
Neospora caninum	0.0399	0.0639	0.0532	0.1388	0.0300	0.0206	0.0293	0.0221	N/A			
Hammondia sp.	0.0400	0.0641	0.0455	0.1312	0.0301	0.0207	0.0293	0.0190	0.0024	0.000001		
Hammondia hammondi	0.0400	0.0681	0.0481	0.1366	0.0301	0.0207	0.0264	0.0221	0.0024	0.0024	N/A	
Toxoplasma gondii	0.0416	0.0701	0.0507	0.1377	0.0301	0.0207	0.0250	0.0236	0.0036	0.0036	0.0012	N/A

[a] Sarcocystidae sp. from American opossums.

[b] Sarcocystidae sp. from Australian opossums.
